# Regionally Distinct Astrocytes Display Unique Transcription Factor Profiles in the Adult Brain

**DOI:** 10.3389/fnins.2020.00061

**Published:** 2020-02-21

**Authors:** Brittney Lozzi, Teng-Wei Huang, Debosmita Sardar, Anna Yu-Szu Huang, Benjamin Deneen

**Affiliations:** ^1^Center for Cell and Gene Therapy, Baylor College of Medicine, Houston, TX, United States; ^2^Program in Developmental Biology, Baylor College of Medicine, Houston, TX, United States; ^3^Department of Neurosurgery, Baylor College of Medicine, Houston, TX, United States

**Keywords:** astrocyte, transcription factor, brain, cellular diversity, bioinformatics

## Abstract

Astrocytes are the most abundant type of glial cell in the central nervous system and perform a myriad of vital functions, however, the nature of their diversity remains a longstanding question in neuroscience. Using transcription factor motif discovery analysis on region-specific gene signatures from astrocytes we uncovered universal and region-specific transcription factor expression profiles. This analysis revealed that motifs for Nuclear Factor-I (NFI) are present in genes enriched in astrocytes from all regions, with NFIB and NFIX exhibiting pan-astrocyte expression in the olfactory bulb, hippocampus, cortex, and brainstem. Further analysis into region-specific motif patterns, identified Nkx3-1, Stat4, Pgr, and Nkx6-1 as prospective region-specific transcription factors. Validation studies revealed that Nkx6-1 is exclusively expressed in astrocytes in the brainstem and associates with the promoters of several brainstem specific target genes. These studies illustrate the presence of multiple transcriptional layers in astrocytes across diverse brain regions and provide a new entry point for examining how astrocyte diversity is specified and maintained.

## Introduction

The brain is composed of an incredible array of diverse cell types, of which, glial cells account for approximately half of this mosaic (Fu et al., 2013; Herculano-Houzel, 2014). Astrocytes, a principal subtype of glial cell, were traditionally thought to be a homogenous population of cells that only function to provide support to neurons. However, astrocytes are now known to perform a multitude of essential functions, such as buffering neurotransmitters, regulating synaptogenesis, modulating synaptic transmission, and maintaining the blood-brain barrier (Abbott et al., 2006). In addition, calcium signaling within astrocytes has been shown to control neuronal physiology and animal behavior (Chai et al., 2017; Yu et al., 2018). Taken together, astrocytes are now known to contribute to nearly every aspect of brain physiology and function (Khakh and Deneen, 2019). Their ability to execute a wide array of functions challenges the notion that astrocytes are a homogenous population of cells.

Astrocytes are electrically silent cells, making it difficult to characterize their functional diversity based on electrophysiological activities. Methods for understanding neuronal cell diversity, such as whole cell electrophysiology (Anderson et al., 2001), morphological criteria, and imaging- based analysis reveals little information about astrocytes because they are not excitable, exhibit grossly uniform (albeit complex) morphologies, and lack subtype-specific markers for imaging (Jobling and Gibbins, 1999; Khakh and Sofroniew, 2015). Critically, the lack of reliable, astrocyte-specific markers has severely hindered the development of tools to study astrocytes. The identification of Aldh1l1 as a marker that broadly and specifically labels astrocytes (Anthony and Heintz, 2007; Cahoy et al., 2008), and the subsequent development of transgenic mice (Anthony and Heintz, 2007) has enabled astrocytes to be isolated and further analyzed. These tools have also enabled further molecular probing of astrocyte transcriptomes, revealing extensive molecular heterogeneity (Bayraktar et al., 2015). Unique astrocytic gene signatures have been found across brain regions (Morel et al., 2017; Duran et al., 2019), and it has been demonstrated that region-specific astrocyte transcriptomes translate to neural-circuit based functional differences (Chai et al., 2017). In addition to regional diversity, five distinct astrocyte sub-populations have been identified across a host of brain regions, and characterization of these sub-populations revealed functional diversity amongst these subpopulations with respect to synapse formation (Lin et al., 2017). While our understanding of astrocyte heterogeneity has advanced considerably, many questions remain about how this intrinsic heterogeneity is encoded, and whether and how these regionally distinct signatures are converted to functional differences.

One important question remaining is what controls the unique expression profiles observed in astrocyte populations from distinct brain regions? In the spinal cord, positionally distinct subpopulations of astrocytes arise from the differential expression of transcription factors during development and this combinatorial transcription factor code results in three distinct astrocyte subpopulations in the developing spinal cord (Hochstim et al., 2008). Applying this rationale to the brain, we hypothesized that differential transcription factor expression contributes to the observed regional diversity of astrocytes in the mature brain. Toward this, we sought to decipher transcription factor expression profiles associated with astrocyte populations by surveying region specific molecular profiles. Bioinformatic analyses of astrocyte gene signatures from the olfactory bulb, hippocampus, cortex, and brainstem identified cohorts of transcription factors involved in modulating region-specific molecular signatures. We identified generalized astrocytic transcriptional regulators, as well as three region-specific transcription factors in adult astrocytes. Our findings suggest that differential expression of transcription factors influences astrocyte diversity in the mammalian brain.

## Materials and Methods

### Animals, Tissue Dissociation, and FACS Analysis

All research and animal care procedures were approved by the Baylor College of Medicine Institutional Animal Care and Use Committee and housed in the Association for Assessment and Accreditation of Laboratory Animal Care-approved animal facility at Baylor College of Medicine. Both male and female BAC Aldh1l1–eGFP mice were used. All strains were maintained on C57BL6 background.

The olfactory bulb, hippocampus, cortex, and brainstem from 16-week old Aldh1l1–eGFP mice was dissected and dissociated using the protocol in Lin et al., 2017. Fluorescence activated cell sorting (FACS) was performed on a BD FACSAria III instrument (100-μm nozzle and 20-p.s.i. setting) with FACSDIVA software, and eGFP+ astrocytes were sorted into a 1.5-ml eppendorf tube containing RLT lysis buffer from the RNeasy Micro Kit (74004, QIAGEN) with 1% ß-Mercaptoethanol.

### Total RNA Extraction, Library Preparation and Sequencing

Total RNA was extracted from Aldh1l1–eGFP+ FAC-sorted cells using the RNeasy Micro Kit (74004, QIAGEN) and quality controlled using the High Sensitivity RNA Analysis Kit (DNF-472-0500, Agilent formerly AATI) on a 12-Capillary Fragment Analyzer. cDNA synthesis, library construction and rRNA depletion was performed on 5 ng total of RNA using the Trio RNA-Seq System (0507-96, NuGEN). The resulting single index libraries were validated using the Standard Sensitivity NGS Fragment Analysis Kit (DNF-473-0500, Agilent formerly AATI) for size confirmation and quantified using the Quant-iT dsDNA Assay Kit, high sensitivity (Q33120, Thermo Fisher). Samples were diluted to equimolar concentrations (2 nM), pooled, and denatured according to the manufacturer’s protocol. The final library dilution of 1.3 pM was sequenced on a NextSeq500 using the High Output v2 kit (FC-404-2002, Illumina) for paired-end (2 × 75) sequencing of approximately 40 million reads per sample.

### Bioinformatics Analysis

Demultiplexed sequencing files were downloaded from BaseSpace and quality control was assessed using fastQC (v0.10.1) and MultiQC (v0.9) (Ewels et al., 2016). Reads were mapped to the mouse genome (10 mm) using STAR (v2.5.0a) (Love et al., 2014). Rsamtools (v2.0.0) and GenomicFeatures (v1.32.2) were used to build count matrices and gene models for expression quantification. UCSC transcripts were downloaded from Illumina iGenomes in GTF file format. We determined reads per million (RPM) using GenomicAlignments (v1.16.0). Principal component analysis (PCA) was performed using rlog transformed gene expression matrix of global gene expression >1 for each region. DESeq2 (v1.20.0) was used for both differential gene expression analysis and read count normalization. Expression heat maps were generated using ComplexHeatmap (v2.0.0).

### Astrocyte Region-Specific Gene Signatures

To identify unique gene signatures, we compared global gene expression from one region to all three other regions using DESeq2. This process was repeated to determine region-specific gene expression patterns. We defined differentially expressed genes (DEGs) as those with normalized reads per million (RPM) >5 in at least two of the replicates and expression fold-change >1.5 at *p* < 0.01. Gene Ontologies associated with region-specific DEGs were determined using Enrichr and visualized using GOplot (v1.0.2) and ggplot2.

### Motif Analysis

To identify any transcription factor motifs that are enriched across region-specific gene signatures, the DEGs from each region were pooled to comprise one list of 3555 genes. These genes were analyzed using Hypergeometric Optimization of Motif EnRichment (HOMER) (v4.10) to identify transcription factor motifs enriched within 2 kb of the gene’s promoter sequence. To be considered enriched across all regions the transcription factor motif had to be present in at least 50% of DEGs from each region. Transcription factors with enriched motifs were further analyzed to determine their expression patterns across regions. Those with an RPM >5 in at least two of the replicates were used for downstream analysis.

Applying the same parameters outlined above, DEGs from each region were individually subjected to motif analysis using HOMER to discover transcription factor motifs enriched within 2 kb of the gene’s promoter sequence. The resulting list of enriched motifs was filtered based on expression data to identify regionally specific transcription factors. We considered a transcription factor regionally enriched only with a fold change >2 at *p* < 0.01 in the region of interest.

### Immunocytochemistry

Perfusion and tissue collection were performed as described previously in Huang et al., 2016. Briefly, mice were deeply anesthetized by isoflurane and then fixed by transcardiac perfusion with PBS followed by 4% PFA in PBS. Tissues for histological analysis were harvested immediately after perfusion. The tissues were then fixed 6 h in 4% PFA in PBS and cryopreserved by overnight incubations in 20% sucrose. Tissues were embedded in OCT compound (Sakura) and sectioned. We collected 30 μm sections of brains with a cryostat and stained them as floating sections. Prewarmed solution of sodium citrate (pH 6.0) was added to immerse the sections, and the sections were incubated in 75°C water bath for 10 min. Sections were allowed to cool down to room temperature and then blocked for 20 min in a PBS solution containing 10% serum (matched to the host used for the secondary antibodies) and 0.3% Triton X-100. Primary antibody incubation was performed in the blocking solution overnight at 4°C for floating sections. Secondary antibody incubation was performed in the PBS solution with 0.1% Triton X-100 for floating sections at room temperature for 1 h. Sections were washed between incubations with PBS containing 0.1% Triton X-100. DAPI was included in the penultimate wash. We used these primary antibodies at the following dilutions: chicken anti-GFP 1:1000 (Abcam, ab13970), mouse anti-Nkx6.1 (DSHB, F55A10), rabbit anti-Pgr 1:200 (Invitrogen, MA5-14505), rabbit anti-NFIB (Millipore Sigma, HPA003956), and rabbit anti-NFIX (Abcam, ab101341). Secondary antibodies conjugated to DyLight 488, 549, or 649 were used at a dilution of 1:500 and raised in goat or donkey (Jackson ImmunoResearch Laboratories). Sections were mounted with antifade mounting medium (VECTASHIELD) and imaged via epifluorescent microscopy (Zeiss M1 with ApoTome2 and ZEN2 software) or Nikon A1-Rs confocal microscope.

### Transcription Factor Target Identification

Potential transcription factor targets were predicted using HOMER’s annotatePeaks.pl with the -m option. Each set of regional DEGs was interrogated for the presence of the associated transcription factor motif allowing zero mismatch. Only genes that had the appropriate motif sequence within 2 kb of the transcriptional start site were considered possible targets. The resulting list was then subset to include only genes with a fold change >2.5 for each region. Predicted target enrichment was visualized using ComplexHeatmap (v2.0.0) and circlize (v0.4.6). Gene Ontologies of predicted targets were determined using Enrichr (Chen et al., 2013; Kuleshov et al., 2016).

### Chromatin Immunoprecipitation (ChIP)

Brainstems were collected from 16-week old mice for ChIP-PCR validation of Nkx6-1 targets. Tissue was coarsely chopped and washed with cold PBS before dissociation with a pellet homogenizer. The homogenate from 6 dissociated brainstems were pooled for subsequent sample preparation. Crosslinking was performed using freshly prepared 1.1% formaldehyde solution (11% formaldehyde, 100 mM NaCl, 1 mM EDTA, 50 mM HEPES pH 7.9) while rocking for 10 min and neutralized by adding glycine (125 mM). Samples were centrifuged at 3500 rpm for 5 min at 4°C, washed with PBS (containing 1 mM PMSF), and pellets were stored at −80°C or lysed immediately. All remaining buffers contain protease inhibitors (Roche Cat. 04693132001). To release nuclei cell pellets were resuspended in PBS/PMSF containing 0.5% Igepal and washed with cold ChIP-Buffer 1 (0.25% Triton-X100, 10 mM EDTA, 0.5 mM EGTA, 10 mM HEPES pH 6.5) and rotated for 10 min at 4°C followed by centrifugation at 1200 rpm for 5 min at 4°C and washing with ChIP-Buffer 2 (200 mM NaCl, 1 mM EDTA, 0.5 mM EGTA, 10 mM HEPES pH 6.5) while rotating at room temperature. Cells were collected via centrifugation and incubated in ChIP lysis buffer (0.5% SDS, 5 mM EDTA, 25 mM Tris–HCl pH 8) for 15–20 min at room temperature to lyse nuclei. Lysates were sonicated to approximately 200–500 bp length fragments using a Bioruptor (Diagenode, model XL). Fragment lengths of chromatin were confirmed using the Standard Sensitivity NGS Fragment Analysis Kit (DNF-473-0500, Agilent formerly AATI) on a 12-Capillary Fragment Analyzer and quantified using Quant-it dsDNA assay kit (Cat. Q33120). An input of 100 μg of sonicated chromatin was used for each experiment, and 1 ug was saved as input chromatin. Samples were diluted 5-fold with ChIP-dilution buffer (1% Triton-X100, 2 mM EDTA, 150 mM NaCl, 20 mM Tris–HCl pH 8) and immunoprecipitation was performed overnight at 4°C with 10 μg of Nkx6-1 antibody (F55410, DSHB) and mouse IgG (Santa Cruz Biotechnology, sc-2025) while rotating. Samples were then incubated with Dynabeads (Invitrogen) for 6 h and purified through a series of washes with TSE1 buffer (0.1% SDS, 1% Triton-X100, 2 mM EDTA, 20 mM Tris–HCl pH 8, 150 mM NaCl), TSE2 buffer (0.1% SDS, 1% Triton-X100, 2 mM EDTA, 20 mM Tris–HCl, 500 mM NaCl), LiCl buffer (0.25 M LiCl, 1% NP-40, 1% sodium deoxycholate, 1 mM EDTA, 10 mM Tris–HCl, pH 8) and TE buffer (10 mM Tris–HCl pH 8, 1 mM EDTA). Samples with beads were then incubated at 65°C for 20 min in ChIP Elution buffer (1% SDS, 0.1 M NaHCO3). ChIP samples and input control were incubated at 55°C with proteinase K (0.2 mg/ml) and NaCl (125 mM) for 3 h followed by overnight incubation at 65°C to reverse crosslinking. Immunoprecipitated DNA was purified using the Qiagen PCR purification kit and analyzed using primers specific to the Hoxc4 and Hoxb3 promoters (Hoxc4 -forward: 5′-GGC CAAGAGGGTTGG, reverse: 5′-GCAGTCTGTGTAGGTCA CAG, Hoxb3-forward: 5′-GCCATTCTGTGTAGACAAGAGC, reverse: 5′-CGGAGAGACGGCTAACAC).

## Results

### Astrocytes Display Brain Region-Specific Gene Expression Signatures

**FIGURE 1:**
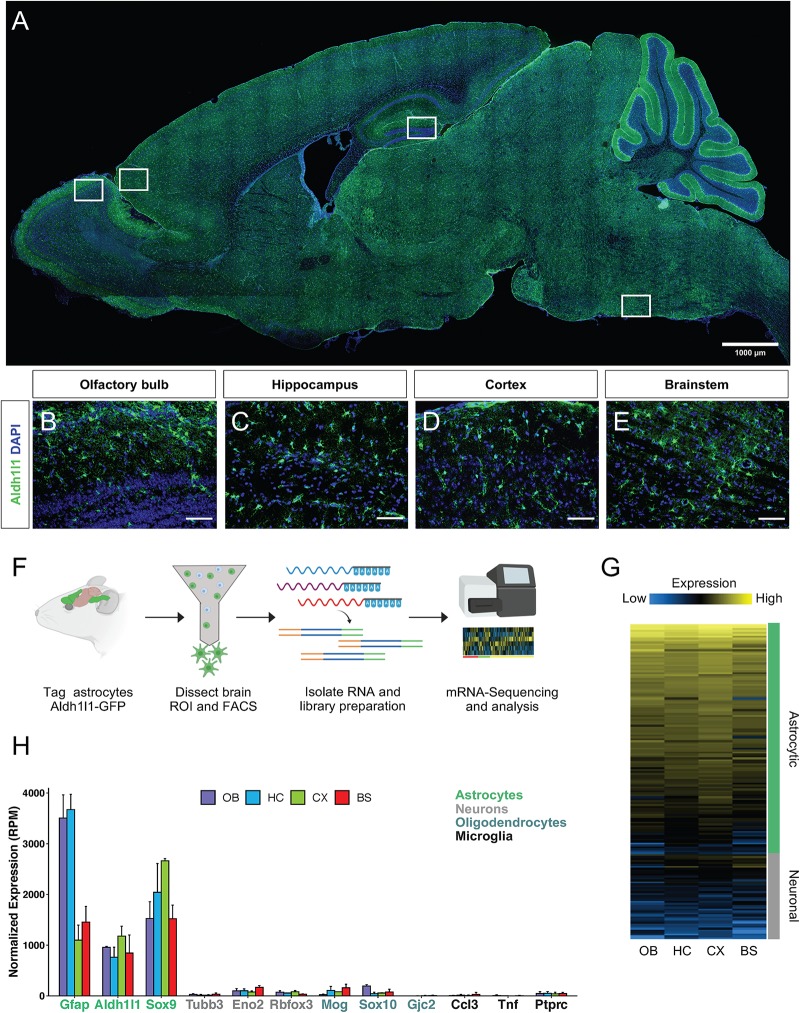
Isolation and verification of Aldh1l1-eGFP astrocytes from selected regions. **(A–E)** Validation of Aldh1l1-eGFP cell specificity through immunofluorescence in, **(A)** whole brain, **(B)** olfactory bulb, **(C)** hippocampus, **(D)** cortex, and **(E)** brainstem. **(F)** Schematic of the approach used to investigate astrocyte regional diversity in the adult mouse brain. **(G)** Validation of cell identity through normalized expression of astrocyte and neuron specific genes. **(H)** Gene expression levels (in reads per million; RPM) of markers for astrocytes, neurons, oligodendrocytes, and microglia. Scale bar = 100 μm. OB, olfactory bulb; HC, hippocampus; CX, cortex; BS, brainstem.

Aldh1l1 is a validated marker of astrocytes (Anthony and Heintz, 2007; Cahoy et al., 2008) and Aldh1l1-eGFP mice have been generated as a tool that broadly, yet specifically, labels astrocytes throughout the brain (Figures 1A–E). To query region specific gene signatures from astrocytes, Aldh1l1-eGFP mice were used to FACS isolate astrocytes from four brain regions (olfactory bulb, hippocampus, cortex, and brainstem) for mRNA-Seq analysis (Figure 1F). We performed whole transcriptome RNA-Sequencing, and to verify that the resultant molecular profiles reflect astrocyte-specific signatures, we compared our data set with existing gene signatures linked to neurons and astrocytes (Lin et al., 2017), finding that our astrocyte expression profiles are consistent with astrocytic-signatures (Figure 1G and Supplementary Datasheet 1). To further confirm that these cells exhibit molecular features exclusive to astrocytes, we examined expression of established markers of astrocytes, neurons, oligodendrocytes, and microglia from our sequencing data (Figure 1H). Together, these data indicate that we have successfully isolated Aldh1l1-eGFP astrocytes and profiled their transcriptomes from distinct brain regions.

**FIGURE 2:**
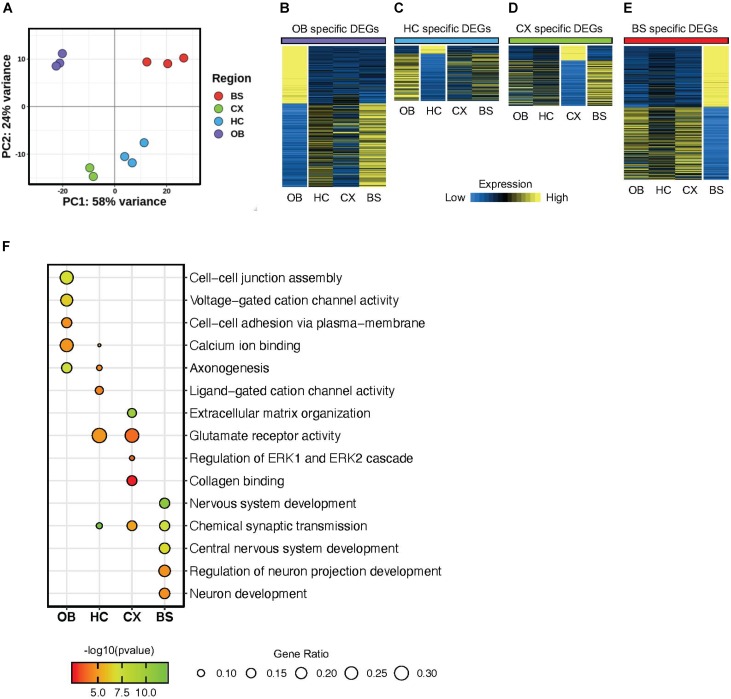
Astrocytes display regionally distinct gene signatures. **(A)** Principal component analysis plot from RNA-Seq results of four brain regions. **(B–E)** Heatmap showing differentially expressed genes from each region **(B)** OB DEGs; purple, **(C)** HC DEGs; blue, **(D)** CX DEGs; green, **(E)** BS DEGs; red *p*-value < 0.01 for all regions. **(F)** Gene Ontology analysis plot of regional differentially expressed genes.

To evaluate the regional diversity of these astrocyte populations, we probed the transcriptome of the four brain regions using various bioinformatics approaches. First, we used principal component analysis (PCA) as an unbiased approach to analyze global gene expression patterns in each region. The PCA revealed distinct gene expression patterns that were unique for each region (Figure 2A). The olfactory bulb (OB) and brainstem (BS) displayed the greatest expression pattern variation, suggesting that astrocytes in the OB and BS are transcriptomically different from astrocytes in other regions. Additionally, the hippocampus (HC) and cortex (CX) exhibited only 19% variability, indicating that astrocytes in the HC and CX share the most similar molecular expression patterns. A similar relationship was also observed between cortical and hippocampal samples in the study from Morel et al., 2017. These results indicate that our independently derived datasets are consistent with previous studies and further support the notion that astrocytes maintain region specific molecular signatures.

Next, we sought to identify region-specific gene signatures. Toward this, we performed differential gene expression analysis by comparing one region to all three other regions to determine region-specific differentially expressed genes (DEGs). The identified DEGs are unique to the region of interest, such that a DEG is significantly up or down regulated only in the respective region when compared to all other regions and exhibits a fold change >1.5 at *p* < 0.01. We found 1360 DEGs in the OB, 398 DEGs in the HC, 505 DEGs in the CX, and 1292 DEGs in the BS (Supplementary Table 1). Visualized in Figures 2B–E we show the expression of each set of DEGs across regions to highlight the enrichment of these DEGs in their respective region. This transcriptome analysis supports observations from the PCA, showing that astrocytes in each of the four regions demonstrate unique, region-specific expression profiles and that the OB and BS are more molecularly distinct than astrocytes from other brain regions. Interestingly, the HC displayed fewer DEGs than any other region, and of those DEGs only 15% were upregulated, while >80% of hippocampal DEGs are downregulated compared to other regions.

Finally, to gain insight into the cellular pathways regulated by these DEGs, we performed gene ontology (GO) analysis on the DEGs from each brain region (Figure 2F). We found that DEGs from each region are involved in an array of diverse biological processes. For instance, the HC DEGs were enriched for ligand-gated cation channel activity, while the DEGs from the OB were associated with cell-cell junction assembly and adhesion. Critically, we also found some enriched biological processes that are conserved across regions including synaptic transmission and glutamate receptor activity. These results indicate that astrocytes from these distinct brain regions exhibit two broad layers of molecular features: conserved and unique.

### Region-Specific Gene Signatures Display Universal Transcription Factor Expression

**FIGURE 3:**
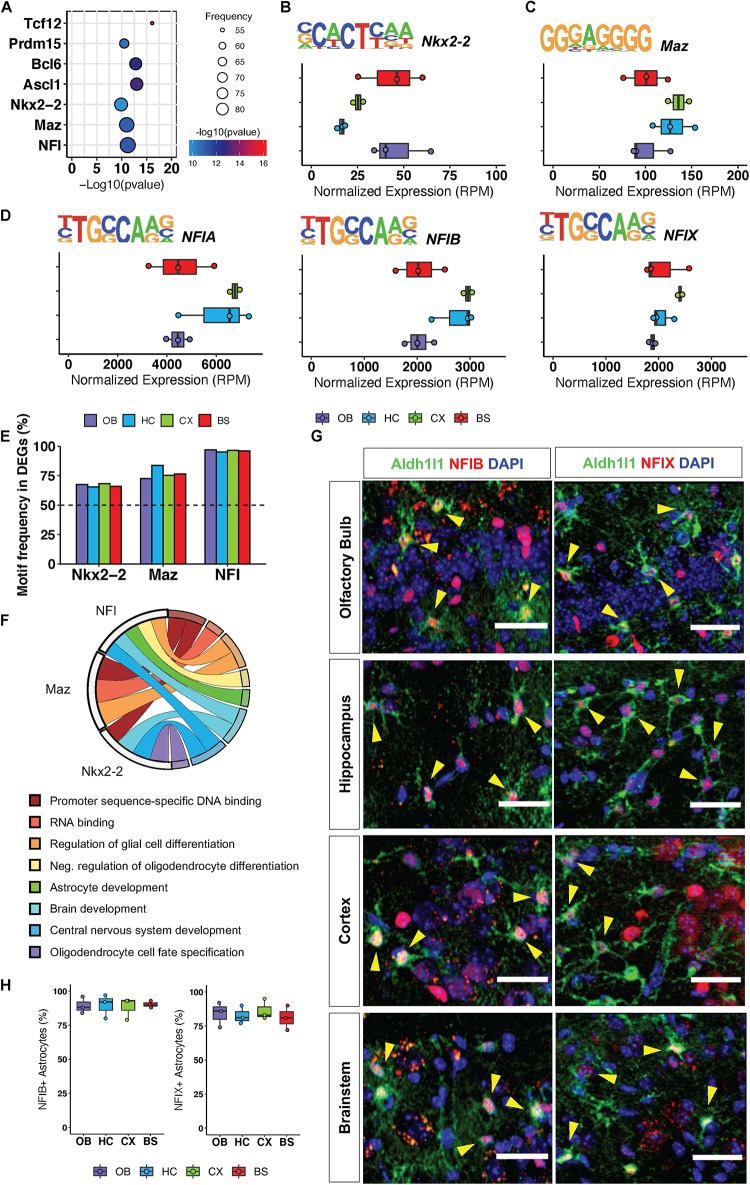
Regional gene signatures exhibit universally conserved transcription factor motif enrichment. **(A)** Significant transcription factor motifs enriched in all regional DEGs. **(B–D)** Motif sequence and normalized expression of top 3 most significantly enriched motifs across surveyed brain regions **(B)** Nkx2-2, **(C)** Maz, **(D)** NFIA/NFIB/NFIX. **(E)** Frequency of motif occurrence in each region DEGs **(F)** Statistically significant Gene Ontologies associated with each transcription factor. *p*-value < 0.05. **(G)** Representative images of immunofluorescence staining of Aldh1l1-eGFP (green) and NFIB or NFIX (red). Yellow arrows indicate double positive cells. **(H)** Quantification of immunofluorescence staining. *n* = 3 scale bar = 50 μm.

To investigate how transcriptional regulation maintains conserved gene ontologies across the brain, we pooled all region-specific DEGs, hypothesizing that since these distinct DEGs are associated with some functionally redundant gene ontologies across the brain, they are likely to be regulated by universally conserved transcription factors. We analyzed the pool of 3555 DEGs from all regions for transcription factor motif enrichment, querying transcription factor binding sites within 2 kb of the 5′ promoter regions of these genes. We found seven transcription factor motifs (RPM >5) that were significantly enriched in DEGs from all four brain regions (Figure 3A), and the top three most significantly enriched motifs were that of Nkx2-2, Maz and NFI-family members NFIA, NFIB, NFIX (Figures 3B–D). Interestingly, these transcription factors have previously been implicated in developmental oligodendrogenesis and gliogenesis (Deneen et al., 2006; Cai et al., 2010; Liu et al., 2016) but have not been studied in adult astrocytes. To ensure equal representation of these transcription factor motifs across DEGs from each region we determined how many genes from each regional signature had an Nkx2-2, Maz, and NFI binding site within 10 kb of the transcriptional start site. The Nkx2-2 motif was identified in 67% of DEGs from each region. The Maz motif was most enriched in the HC DEGs but was still present in 77% of DEGs from each of the other three regions. The NFI motif was the most frequently identified of the three transcription factors in all the regions with its binding sequence appearing in at least 96% of DEGs from each region (Figure 3E). We also determined GO categories associated with the genes containing these universally conserved transcription factor binding sites to gain insight into the pathways or biological processes through which they may act. As expected, genes containing these motifs are found in ontology categories centralized around brain development and astrocyte function (Figure 3F).

Our *in silico* analysis suggests that NFI transcription factors operate in mature astrocytes to regulate the expression of key genes associated with synaptic physiology. Moreover, NFI transcription factors exhibit a significantly higher motif frequency in all regional DEGs; therefore, we set out to validate expression of the NFI family members in adult astrocytes from all four regions. Since NFIA is known to be expressed in adult astrocytes (Laug et al., 2019), we sought to confirm expression of the other NFI family members: NFIB and NFIX. Using immunohistochemistry, we found that NFIB and NFIX co-localize with Aldh1l1-eGFP astrocytes in all four brain regions (Figure 3G). We find that NFIB and NFIX expression is equally widespread across all four brain regions and can be found in approximately 86% of Aldh1l1-eGFP expressing astrocytes throughout the brain (Figure 3H). Furthermore, consistent with previous findings (Chen et al., 2017), we also observed expression of NFIB and NFIX in neurons, though not to the same extent as their astrocytic expression. Taken together, these analyses suggest the presence of conserved transcription factor programs across diverse brain regions that function to maintain expression of genes that regulate core astrocytic functions.

### Astrocytes Exhibit Region-Specific Transcription Factor Signatures

**FIGURE 4:**
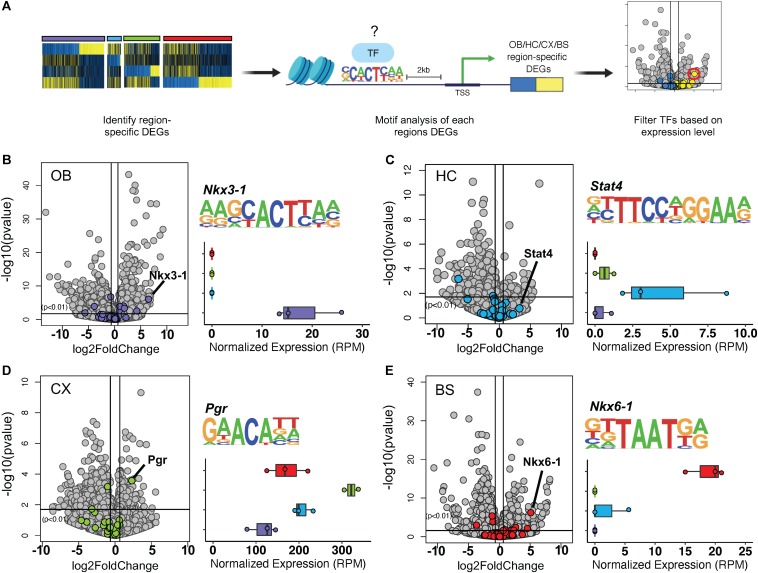
Transcription factor motif enrichment is regionally distinct. **(A)** Schematic of bioinformatic approach to identify region-specific transcription factors. **(B–E)** Volcano plot showing significance versus log2 fold change. Transcription factor motifs enriched in each region’s DEGs colored. Most upregulated statistically significant transcription factor motif sequence and normalized expression shown. **(B)** Nkx3-1 in OB, **(C)** Stat4 in HC, **(D)** Pgr in CX, **(E)** Nkx6-1 in BS. *p*-value < 0.01.

Thus far, our observations indicate the existence of transcription factors that are universally expressed in nearly all adult astrocytes, across a host of diverse brain regions. Given that transcription factor patterning has been shown to define distinct regional expression profiles in the spinal cord (Liu et al., 2003; Hochstim et al., 2008) and because our cross-region comparisons identified unique molecular profiles for each brain region (Figures 2B–E), we next sought to determine whether astrocytes from these distinct regions also exhibit unique transcription factor expression profiles. Toward this, we first analyzed the DEGs from each region individually for transcription factor motif enrichment. After compiling a list of known motif sequences enriched in each region, we filtered the list so that only significantly upregulated transcription factors were considered to determine which, if any, were enriched in only one region (Figure 4A). Using a fold change threshold of 2 at *p* < 0.01 we were able to identify uniquely enriched transcription factors in the OB, CX, and BS (Figures 4B–E). The HC did not show any significant transcription factor enrichment owing to the fact that it did not exhibit a robust DEG profile (Figure 4C). In the OB and BS, several transcription factors were significantly upregulated, but only the most highly upregulated transcription factor was used for downstream networking analysis and validation. We found Nkx3-1 in the OB (Figure 4B) and Nkx6-1 in the BS (Figure 4E), both of which were almost exclusively expressed in the region of interest. In the CX, we found that Pgr was detectable in all four regions but was expressed more than 4-fold in the CX compared to other regions (Figure 4D). These results suggest that astrocytes exhibit region-specific expression of transcription factors.

**FIGURE 5:**
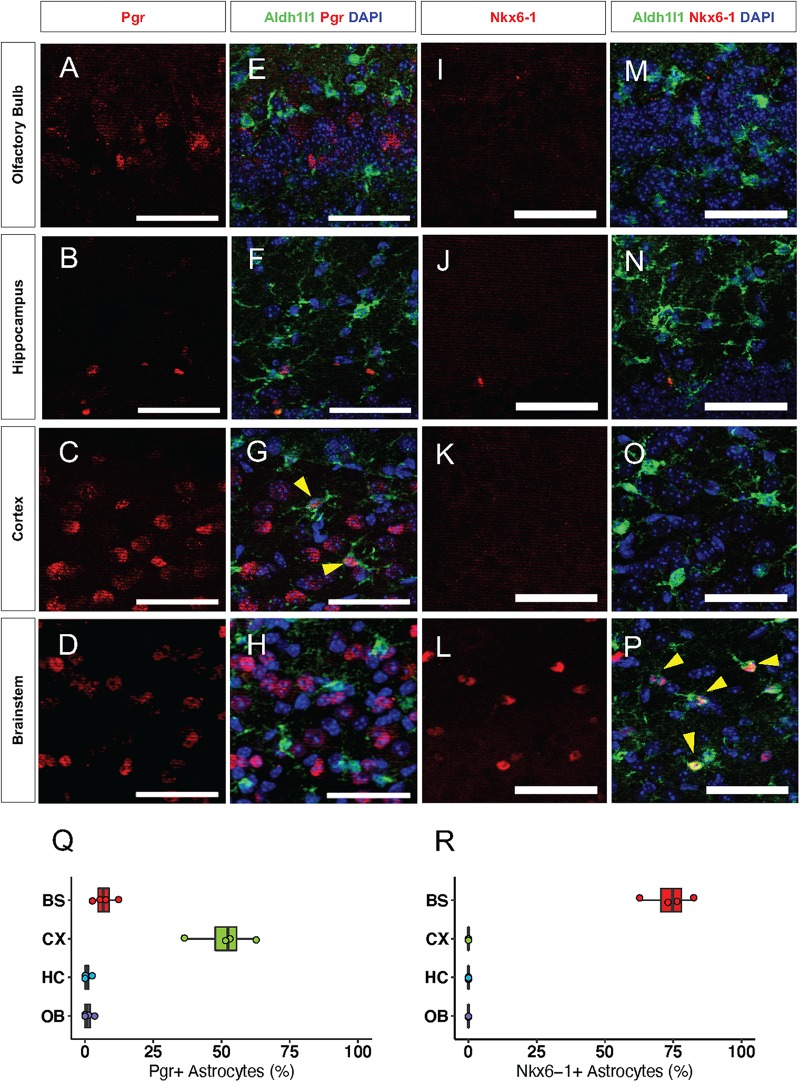
Transcription factors are regionally specific *in vivo*. **(A–H)** Representative images of Immunofluorescence staining of Pgr (red) in the **(A)** OB, **(B)** HC, **(C)** CX, and **(D)** BS. **(I–P)** Antibody staining of Nkx6-1 (red) in the **(I)** OB, **(J)** HC, **(K)** CX, and **(L)** BS. **(E–H)** and **(M–P)** are the same panels as in **(A–D)** and **(I–L)**, respectively, but include Aldh1l1-eGFP expression (green). Yellow arrows indicate double positive cells. **(Q–R)** Quantification of immunofluorescence staining. **(Q)** Pgr and **(R)** Nkx6-1. *n* = 4 Scale bar = 50 μm.

Next, we sought to validate our bioinformatics analyses by performing immunolabeling with antibodies to candidate transcription factors on brains from 16-week old Aldh1l1-eGFP mice. We found that the CX-specific transcription factor, Pgr, was expressed in neurons throughout the brain. However, Pgr co-localized with 51% (Figure 5Q) of Aldh1l1-eGFP expressing astrocytes only in the CX (Figures 5A–H), supporting our hypothesis that Pgr may regulate astrocytic molecular profiles in the CX. Antibody staining of Nkx6-1 revealed that it is expressed exclusively in the BS, where it co-localizes with 74% of Aldh1l1-eGFP astrocytes (Figures 5I–P,R). Interestingly, not all astrocytes expressed Pgr or Nkx6-1 in their respective regions, likely due to additional layers of local diversity (Lin et al., 2017). These results, in conjunction with our validation studies on NFI-family members, indicate that astrocytes exhibit both universal and region-specific transcription factor expression profiles.

### Predicted Targets of Regional Transcription Factors

**FIGURE 6:**
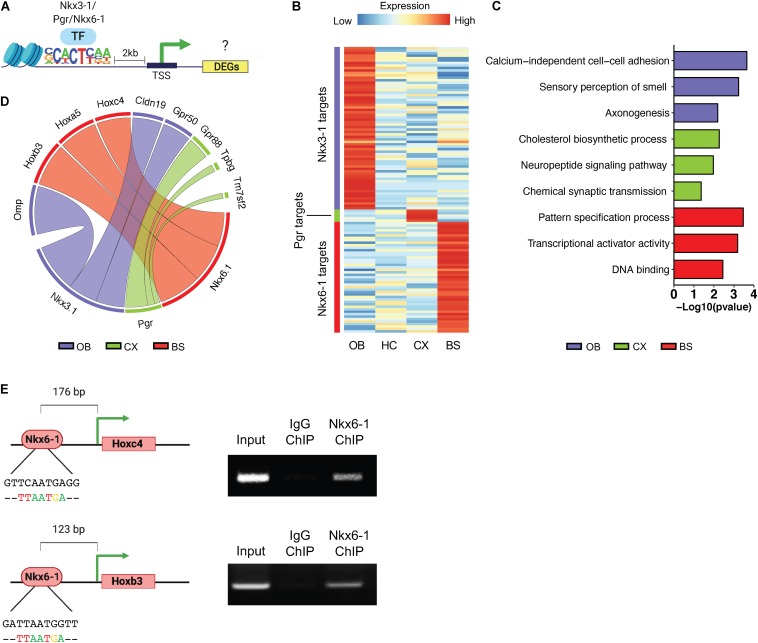
Transcriptional regulatory network profiling. **(A)** Schematic of methodology for predicting transcription factor targets. **(B)** Heatmap showing log2 transformed expression of potential transcription factor targets. **(C)** Gene ontology analysis of predicted targets. **(D)** Circos plot to visualize top predicted targets by transcription factor, line width represents fold change. **(E)** ChIP-PCR Schematic and ChIP-PCR result of Nkx6-1 binding at Hoxc4 and Hoxb3 promoters in the brainstem.

It has been suggested that astrocytes maintain regional heterogeneity to afford them specialized functions for interacting with neurons in their specific regional circuitry units (Chung et al., 2015; Hasel et al., 2017). Region-specific transcription factor profiles may be a means to orchestrate these specialized profiles, therefore, we set out to investigate the region-specific transcriptional networks controlled by the above-mentioned transcription factors. Toward this, we sought to identify potential targets of the region-specific transcription factors identified above. First, we further curated the region-specific signatures, focusing on the location of the region-specific transcription factor motif sequence using HOMER, and relative expression of a given DEG (Figure 6A). A gene was considered a target if it had an instance of the motif sequence within 2 kb of the transcription start site and was significantly upregulated by at least a 2.5-fold at *p* < 0.01. Using this approach, we identified 66 predicted targets of Nkx3-1 in the OB, 45 for Nkx6-1 in the BS, and only 5 for Pgr in the CX. As there were no significantly enriched transcription factors identified in the HC it was not considered in subsequent analysis. The log2 transformed RPM of each transcription factor’s targets are visualized in a heatmap (Figure 6B).

Next, to determine what biological pathways these potential targets were associated with we performed GO analysis (Figure 6C). Targets of each transcription factor were associated with distinct GO categories such as sensory perception of smell in the OB and pattern specification process in the BS. To determine if these regional transcription factors are actively regulating potential targets, we chose the top 3 most likely candidates from each region shown in Figure 6D. Since Nkx6-1 showed the most specific expression pattern in mouse brain (Figures 5L,P), we sought to validate the top targets of Nkx6-1 in the BS by ChIP-PCR. Among the top 3 most likely candidates, Hoxc4 and Hoxb3 were found to have a Nkx6-1 binding motif at their proximal promoters. We collected the BS from 16-week old mice, performed chromatin immunoprecipitation (ChIP) with Nkx6-1, and PCR amplified regions with the Nkx6-1 motif (Figure 6E). We confirmed binding of Nkx6-1 at the promoters of Hoxc4 and Hoxb3 in the adult BS (Figure 6E). Taken together, these data provide additional support that our bioinformatic pipeline can identify region-specific transcription factors that are active in manipulating the regional molecular landscape of astrocytes.

## Discussion

### A Bioinformatic Approach to Study Astrocyte Heterogeneity

The molecular heterogeneity of astrocytes across diverse brain regions has been profiled extensively in recent years, but a clear understanding of the mechanisms that give rise to their vast diversity has long eluded us. In the present study we hypothesized that differential expression of transcription factors controls the region-specific molecular signatures observed in astrocytes across the brain. To test this, we analyzed the astrocyte transcriptomes across four brain regions to first establish DEGs in each region of interest that constitute region-specific gene signatures. Further analysis of region-specific DEGs revealed functionally redundant gene ontologies are associated with the unique gene profiles from each region. Together, these observations suggest that core astrocyte functions are achieved through distinct molecular mechanisms. This prompted us to search these signatures for transcription factors whose expression is conserved across all region-specific DEGs, as these transcription factors may regulate functionally redundant gene ontology pathways. Critically, we find the motif sequence of the NFI family of transcription factors enriched in DEGs and ubiquitous expression of NFIB/NFIX in astrocytes from all four regions (Figure 3). Finally, we identified transcription factors in the OB, CX, and BS whose expression is enriched in only the region of interest to regulate region-specific pathways in astrocytes. These results present a new bioinformatic approach to study astrocyte diversity through the lens of transcription factors and the essential regulatory mechanisms they offer.

### Unique and Conserved Transcription Factors Regulate the Astrocyte Transcriptome

Transcriptomic analyses have indeed revealed unique region-specific astrocyte signatures that translate to spatially distinct functional differences (Chai et al., 2017; Morel et al., 2017). However, an important question remains regarding how regionally distinct astrocytes in the brain are endowed with these unique molecular and functional features. One explanation is that a homogenous population of astrocytes migrate throughout the brain during development, and after reaching their final location they develop region-specific molecular and function distinctions. Toward this, it has been suggested that astrocytes undergo molecular reorganization upon terminal migration to become specialized for interacting with neighboring neurons in their specific region (Molofsky et al., 2014; Chai et al., 2017). Indeed, studies have shown that astrocytes from different regions uniquely modify their molecular signatures upon loss of neuronal glutamatergic signaling (Morel et al., 2014) or activation of sonic hedgehog signaling from neighboring neurons (Farmer et al., 2016).

Another possibility is that these diverse features of astrocytes are developmentally pre-ordained, where molecularly distinct subpopulations of astrocytes are specified, and each subtype migrates to different locations where they maintain region-specific heterogeneity into adulthood. In support of this mechanism, it has been shown that astrocyte spatial identity (Tsai et al., 2012) and heterogeneity (Morel et al., 2017) is intrinsically defined by early embryonic dorsoventral axis patterning. Additionally, a combinatorial code involving differential expression of transcription factors during development was shown to specify astrocyte positional identity which results in distinct populations of astrocytes in the spinal cord (Hochstim et al., 2008). Whether astrocyte heterogeneity is specified in conjunction with developmental patterning or cultivated later according to regional circuitry requirements remains to be determined, but undoubtably transcriptional regulation plays a role in the complex quandary of astrocyte diversity.

Here, we ask if a transcription factor code can be defined for maintaining regionally distinct astrocyte populations. By interrogating region-specific DEGs our analysis revealed Nkx2-2, Maz and NFI family members as transcription factors that are universally conserved across the brain and regulate functionally redundant gene ontologies. Nkx2-2 is known to repress neurogenesis to promote oligodendrocyte precursor cell differentiation (Zhou et al., 2001). Nkx2-2 does not co-localize with the astrocytic marker GFAP (Qi et al., 2001), and enrichment of the its motif in adult astrocytes suggests that it likely represses these astrocytic genes during oligodendrocyte development. The second conserved regulator, Maz, has been shown to stimulate gliogenesis *in vitro* by regulating Notch signaling (Liu et al., 2016). Here, we chose to focus on the third regulator, the NFI family of transcription factors because its conserved motif sequence occurs most frequently in all regional gene profiles. Previously, NFIA and NFIB have been shown as necessary and sufficient to initiate gliogenesis (Deneen et al., 2006). Less is known about the role of NFIX in glia, although it has been suggested that NFIB can activate NFIX after the gliogenic switch to regulate terminal glial differentiation in the spinal cord (Matuzelski et al., 2017). Despite being characterized during developmental gliogenesis we find expression of all NFI family members in adult astrocytes. These data suggest a continued importance of gliogenic fate determinants in adult astrocytes, and it warrants further investigation.

Additionally, our data suggests that Nkx3-1, Pgr, and Nkx6-1 may act as region-specific transcription factors to regulate the unique molecular profiles observed in astrocytes across regions. The HC had the fewest DEGs compared to the other regions, making it more difficult to identify enriched transcription factors. However, the identification of region-specific transcription factors in the OB, CX, and BS suggests that the unique molecular signatures identified in each region may be maintained by Nkx3-1, Pgr, and Nkx6-1, respectively. Nkx3-1 expression was reported in the OB, but it has not been studied in astrocyte function or maintenance (Tanaka et al., 1999). Previous studies have shown that Pgr regulates Nrg1 to modulate synaptic activity and synaptogenesis in astrocytes (Lacroix-Fralish et al., 2006). It is widely accepted that Nkx6-1 is involved in specification (Zhao et al., 2014), patterning (Liu et al., 2003) and astrocyte positional identity (Hochstim et al., 2008) in the developing spinal cord. A role for Nkx6-1 in development has been well defined, but it has not been studied in adult astrocytes. Thus, we further validated the expression of Pgr and Nkx6-1 *in vivo* and found that Pgr is expressed in neurons throughout the brain, but colocalizes with astrocytes only in the cortex while Nkx6-1 expression is exclusive to the BS where it labels most astrocytes. These data supported our hypothesis that astrocyte regional heterogeneity is maintained by region-specific transcription factors. Furthermore, the region-specific expression of a developmental patterning factor suggests astrocyte diversity may be intrinsically specified during development and it merits investigation in future studies.

### The Transcriptional Networks That Modulate Astrocyte Diversity

While understanding how astrocyte diversity is specified and maintained is important it is also critical to determine why they display such broad diversity. One likely explanation is that astrocyte diversity enables specialized interactions between astrocytes and the neuronal circuits of their spatial domain (Tsai et al., 2012). Indeed, when comparing astrocyte involvement in neural circuits from the hippocampus and striatum differences were observed between the two regions in potassium buffering, glutamate recycling, and calcium signaling, among others (Chai et al., 2017). These data highlight the importance of defining the transcriptional networks regulated by region specific transcription factors and translating molecular data into functional profiles of astrocyte heterogeneity.

To investigate the regulatory networks that may be controlled by these region-specific transcription factors we determined which genes they are regulating by predicted targets of Nkx3-1, Pgr, and Nkx6-1 in their respective brain regions. Nkx3-1 and Nkx6-1 had the largest number of potential targets and because Nkx6-1 showed the most exclusive region-specific expression in the BS, we confirmed direct regulation of one of its targets, Hoxc4 and Hoxb3, using ChIP-PCR. It is well documented that Hox genes follow distinct regional expression patterns and have been implicated in dorso-ventral patterning of the spinal cord and hindbrain (Gaufo et al., 2004; Di Bonito et al., 2013a). The role of Hox genes in the adult brain is not well understood, although studies show their expression can be detected in various regions throughout the adult brain (Hutlet et al., 2016). Given the specific expression patterns of Hox genes observed both during development (Di Bonito et al., 2013b) and in adulthood (Hutlet et al., 2016) these targets of Nkx6-1 hint at multilayered transcription factor regulation to control regional astrocyte diversity.

Our discovery of Nkx6-1 as a region-specific transcription factor in the adult brain, coupled with its established role in developmental patterning of astrocytes in the spinal cord, suggests that astrocytes in the brain are also subject to transcriptionally regulated patterning. These prospective patterning mechanisms could contribute to molecularly and functionally diverse populations of astrocytes throughout the brain. In summary, our study not only provides evidence of a region-specific transcription factor code through the identification of Nkx6-1, but also opens the door to identifying and characterizing astrocytic patterning transcription factors in the brain. In addition, our study defines a new approach to study astrocyte diversity by interrogating transcription factor profiles to provide insights into region-specific gene regulatory networks across the brain.

## Data Availability Statement

The datasets generated for this study can be found in the GEO repository, accession GSE143282.

## Ethics Statement

The animal study was reviewed and approved by the Baylor College of Medicine IACUC.

## Author Contributions

BL and BD conceived the project. T-WH performed all the immunostaining. DS assisted with the writing and ChIP assay. AH bred the mice and assisted with FACS. BL performed all bioinformatics analysis.

## Conflict of Interest

The authors declare that the research was conducted in the absence of any commercial or financial relationships that could be construed as a potential conflict of interest.
